# Hepatic expression of lipopolysaccharide-binding protein (*Lbp*) is induced by the gut microbiota through *Myd88* and impairs glucose tolerance in mice independent of obesity

**DOI:** 10.1016/j.molmet.2020.100997

**Published:** 2020-04-16

**Authors:** Antonio Molinaro, Ara Koh, Hao Wu, Marc Schoeler, Maria Ilaria Faggi, Alba Carreras, Anna Hallén, Fredrik Bäckhed, Robert Caesar

**Affiliations:** 1 The Wallenberg Laboratory, Department of Molecular and Clinical Medicine, University of Gothenburg, 41345, Gothenburg, Sweden; 2 Department of Molecular Cell Biology, Samsung Biomedical Research Institute, Samsung Medical Center, School of Medicine, Sungkyunkwan University (SKKU), Suwon, 16419, Republic of Korea; 3 Novo Nordisk Foundation Center for Basic Metabolic Research, Section for Metabolic Receptology and Enteroendocrinology, Faculty of Health Sciences, University of Copenhagen, 2200, Copenhagen, Denmark; 4 Region Västra Götaland, Sahlgrenska University Hospital, Department of Clinical Physiology, Gothenburg, Sweden

**Keywords:** Gut microbiota, MYD88, Lipopolysaccharide-binding protein, Glucose metabolism, Liver, CRISR-CAS9

## Abstract

**Objective:**

Gut-derived inflammatory factors can impair glucose homeostasis, but the underlying mechanisms are not fully understood. In this study, we investigated how hepatic gene expression is regulated by gut colonization status through myeloid differentiation primary response 88 (MYD88) and how one of the regulated genes, lipopolysaccharide-binding protein (*Lbp*), affects insulin signaling and systemic glucose homeostasis.

**Methods:**

Liver transcriptomics analysis was conducted on four groups of mice fed a chow diet: conventionally raised (CONV-R) wild-type, germ-free (GF) wild-type, CONV-R *Myd88* KO, and GF *Myd88* KO. Primary hepatocytes were exposed to combinations of lipopolysaccharide (LPS), LBP, and the LBP-blocking peptide LBPK95A, and the effect on insulin signaling was determined. To assess how LBP affects glucose metabolism *in vivo*, two mouse models were applied: treatment with LBPK95A and hepatic knockdown of *Lbp* using CRISPR-CAS9.

**Results:**

We showed that the colonization status regulates gene expression in the liver and that a subset of these genes, including *Lbp*, is regulated through MYD88. Furthermore, we demonstrated that LBP impairs insulin signaling in hepatocytes in the presence of low levels of LPS and that the effect of LBP is abolished by LBPK95A. We showed that both systemic pharmacological blocking of LBP by LBPK95A and CRISPR-CAS9-mediated downregulation of hepatic *Lbp* improve glucose homeostasis.

**Conclusions:**

Our results demonstrate that the gut microbiota regulates hepatic expression of *Lbp* through MYD88-dependent signaling. LBP potentiates LPS inhibition of insulin signaling *in vitro* and impairs systemic glucose homeostasis *in vivo*.

## Highlights

• Gut microbiota induces hepatic expression of *Lbp* through MYD88.
• LBP impairs insulin signalling in hepatocytes in the presence of low levels of LPS.
• Pharmacological blocking of LBP improves systemic glucose homeostasis.
• Knockdown of hepatic *Lbp* improves systemic glucose homeostasis.

## Abbreviations

GF: germ free
CONV-R: conventionally raised
MAMP: microbe-associated molecular pattern
LPS: lipopolysaccharide
KO: knockout
TLR: Toll-like receptor
MYD88: myeloid differentiation primary response 88
TRIF: TIR-domain-containing adapter-inducing interferon-β
LBP: lipopolysaccharide-binding protein
IU: infectious units
ANOVA: analysis of variance
Saa2: serum amyloid A2
Atp11b: probable phospholipid-transporting ATPase IF
Adgrf: adhesion G protein-coupled receptors
Hsd3b2: hydroxy-delta-5-steroid dehydrogenase, 3 beta- and steroid delta-isomerase 2

## 1 Introduction

The gut microbiota is an important regulator of host metabolism in health and disease [1] The influence of the gut microbiota on host metabolism has been extensively studied using germ-free (GF) mice, which display a better metabolic phenotype than conventionally raised (CONV-R) mice, both on a chow diet and during metabolic challenges such as a Western diet [[2], [3], [4]].

The gut microbiota influences host metabolism through several mechanisms. Bacterially produced metabolites interact with host receptors involved in metabolic control, both in the gut and peripheral organs [[5], [6], [7]]. Microbe-associated molecular patterns (MAMPs) are microbially produced metabolites with conserved molecular motifs recognized by the innate immune system. MAMPs that are transferred from the gut into the body can activate inflammatory pathways and may contribute to the development of pathophysiological conditions such as impaired glucose metabolism [4,[8], [9], [10]]. Toll-like receptors (TLRs) are a group of cellular receptors recognizing MAMPs. Most TLRs require the adaptor molecule myeloid differentiation primary response 88 (MYD88) to activate downstream targets [11]. TLRs and MYD88 are expressed in immune cells such as macrophages as well as in many other cell types including hepatocytes [12,13]. Consumption of a Western-style diet, obesity, and impaired glucose homeostasis are associated with increased plasma levels of TLR ligands such as lipopolysaccharide (LPS) [8,14], but LPS is also present in the plasma of healthy human subjects [15] and in lean mice fed a chow diet [4,8]. However, it is unclear at present whether LPS impairs glucose metabolism independent of obesity.

In this study, we conducted transcriptome profiling on liver tissue from GF and CONV-R wild-type and *Myd*88 knockout (KO) mice fed a chow diet to investigate how interactions between the gut microbiota and the immune system influence the expression of genes related to glucose metabolism. We also investigated how hepatic lipopolysaccharide-binding protein (LBP), which we found to be induced by the gut microbiota through MYD88, affects insulin signaling and systemic glucose homeostasis.

## 2 Materials and methods

### 2.1 Mouse experiments

Tissue samples for transcriptome profiling were harvested from male C57Bl6/J mice maintained under CONV-R or GF conditions as described in a previously published study [16]. Four groups of mice were included: CONV-R wild-type, GF wild-type, CONV-R *Myd88* KO, and GF *Myd88* KO. The mice were maintained on a 12-h cycle (light from 7 am to 7 pm) and fed an autoclaved chow diet (LabDiet, St. Louis, MO, USA) and water *ad libitum*. The mice were kept in cages with 3–5 mice in each cage. The GF mice were maintained in flexible film isolators. The mice were killed by cervical dislocation after 4 h of fasting. The *Myd88* KO mice were backcrossed at least eight generations to C57Bl6/J and the last two crossings were conducted using mice from our colony and thereafter separated by a maximum of two generations. The GF and CONV-R mice were separated by a maximum of three generations.

To deplete the gut microbiota by antibiotics treatment, 100 μg/mL neomycin, 50 μg/mL streptomycin, 100 U/ml ampicillin, 50 μg/mL vancomycin, 100 μg/mL metronidazole, 1 mg/mL bacitracin, 125 μg/mL ciprofloxacin, and 100 μg/mL ceftazidime were administered in the drinking water *ad libitum* and replaced with freshly prepared cocktails three times a week. The mice were treated with antibiotics for five weeks.

To study how interactions between LPS and LBP affect systemic glucose homeostasis *in vivo*, wild-type CONV-R mice were administrated 3 intraperitoneal injections for 24 h (time point 0, 12 and 24 h) with the LBP-blocking peptide LBPK95A [17] (5 mg/kg; sequence: RVQGRWKVRASFFK; GeneScript, Piscataway, NJ) or 0.9% sodium chloride the day before the glucose tolerance test.

To knock out *Lbp in vivo*, the mice were injected with 3x10^9^ infectious units (IU) Ad5-CMV-CAS9 and 3 × 10^9^ IU Ad5-U6-sgRNA-*Lbp* retro-orbitally. Mice injected with 6 × 10^9^ IU Ad5-CMV-CAS9 were used as negative controls (Sirion, Planegg, Germany). A glucose tolerance test was conducted after 5 weeks and the mice were killed after 7 weeks. To assess the efficiency of the knockout, proteins were extracted from the liver and epididymal adipose tissue and LBP were quantified by Western blotting.

The animal protocols were approved by the Research Animal Ethics Committee in Gothenburg.

### 2.2 CRISPR/CAS9 construct

A series of three candidate oligonucleotide guide cassettes targeting exon 1 of the *Lbp* gene were cloned into the plasmid pENTR-U6-tracRNA and verified by sequencing. To test the efficiency of the sgRNA sequence, a DNA fragment containing the target sites of the sgRNAs to be tested was cloned out of frame to the N-terminus of the eGFP coding region in plasmid pENTR-CMV-MCS-eGFP. Reporter cells were transfected with the vectors pENTR-U6-sgRNA-LBP (guide), pENTR-CMV-spCAS9 (CAS9), and pENTR-CMV-LBP-target-sites-eGFP (reporter). A strand break at the target site in front of the GFP gene resulting in a frameshift mutations and subsequent expression of GFP indicated the construct's efficiency. The guide sequence CGGTGTCAACCCCGGTGTGG efficiently induced GFP and the U6-sgRNA-LBP-cassette containing this sequence was recombined into an Ad5 vector. Cloning success was verified by restriction analysis and DNA sequencing. Amplification was conducted in HEK293 cells and viral particles were purified via CsCl_2_ purification.

### 2.3 Quantitative PCR

Quantitative PCR (qPCR) to enumerate bacterial 16S rRNA gene copies in the genomic DNA extracted from cecal samples was conducted as previously described [18]. The total amount of bacterial DNA was quantified with the universal primers UniF (5′ GTGSTGCAYGGYYGTCGTCA-3′) and UniR (5′-ACGTCRTCCMCNCCTTCCTC-3′) using the 16S rRNA gene of *Escherichia coli* W3310 as standard.

Determination of the relative gene expression in the primary hepatocytes and liver tissue was conducted by qPCR as previously described [3]. The gene expression data were normalized to the ribosomal protein L32. The primer sequences used in this study were L32F (CCTCTGGTGAAGCCCAAGATC), L32R (CTGGGTTTCCGCCAGTTT), *Lbp*F (GATCACCGACAAGGGCCTG), and *Lbp*R (GGCTATGAAACTCGTACTGCC).

### 2.4 RNA isolation, microarray processing, and statistical analysis

RNA isolation and microarray processing were conducted as previously described [16] Briefly, liver RNA was isolated using an RNeasy Mini Kit (Qiagen, Hilden, Germany). The RNA concentration and quality were evaluated by spectrophotometric analysis (ND-1000; NanoDrop Technologies, Wilmington, DE, USA) and capillary electrophoresis on a 2100 Bioanalyzer (Agilent Technologies, Santa Clara, CA, USA).

RNA labeling, microarray hybridization, and scanning were conducted at the Uppsala array platform core facility at Uppsala University using MoGene 1.0 ST chips (Affymetrix, Santa Clara, CA, USA) according to the manufacturer's instructions. Normalization and probe set summarization were conducted using Affymetrix expression console software. Genes were annotated against the ENSEMBL17 set of genes using the MoGene 1.0 ST probe set mapping provided using BioMart [19]. Overall, 22,398 unique genes were obtained and then normalized by the robust multi-array average method (background-adjusted, normalized, and log-transformed) using the oligo package [20,21] for further downstream data analyses. A hierarchical modeling approach combining generalized linear regression and empirical Bayes statistics within the limma package (version 3.34.9) [22] was used to access the differential gene expression with the following formula: microbiota + genotype + interaction. Multiple testing correction was adjusted by the default Benjamini-Hochberg method [23]. The normalized abundance table for those differentially expressed genes were used for principal component analysis (PCA). Microarray data for wild-type mice were partly analyzed in a previous report [24]. CEL files and normalized data were deposited into the NCBI GEO repository (accession number GSE31115).

### 2.5 Preparation of primary hepatocytes and *in vitro* experiments

Primary mouse hepatocytes were isolated by perfusing the liver with collagenase type IV as previously described [25]. After perfusion, 1.6 × 10^6^ cells were plated on collagen-coated 60 mm dishes in Dulbecco's modified Eagle's medium (DMEM)/F12 (Thermo Fisher) supplemented with fetal bovine serum (Thermo Fisher), penicillin/streptomycin, and 100 nM dexamethasone (Sigma Aldrich). After 4 h, the medium was changed to DMEM/F12 containing penicillin/streptomycin. After 14 h, primary hepatocytes were cultured in DMEM/F12 with or without LPS (diluted with water), LBP (R&D Systems, 870-LP-025, diluted with 0.1% BSA in PBS), or LBPK95A (diluted with 0.1% BSA in PBS) for 8 h and treated with 5 nM insulin for the times indicated in Figure 3. The primary mouse hepatocytes in the experiment presented in Figure 2C were treated with LPS or supernatant from RAW 264.7 (pretreated with LPS for 24 h) for 24 h. RAW 264.7 macrophages were cultivated as previously described [4].

**Figure 1:**
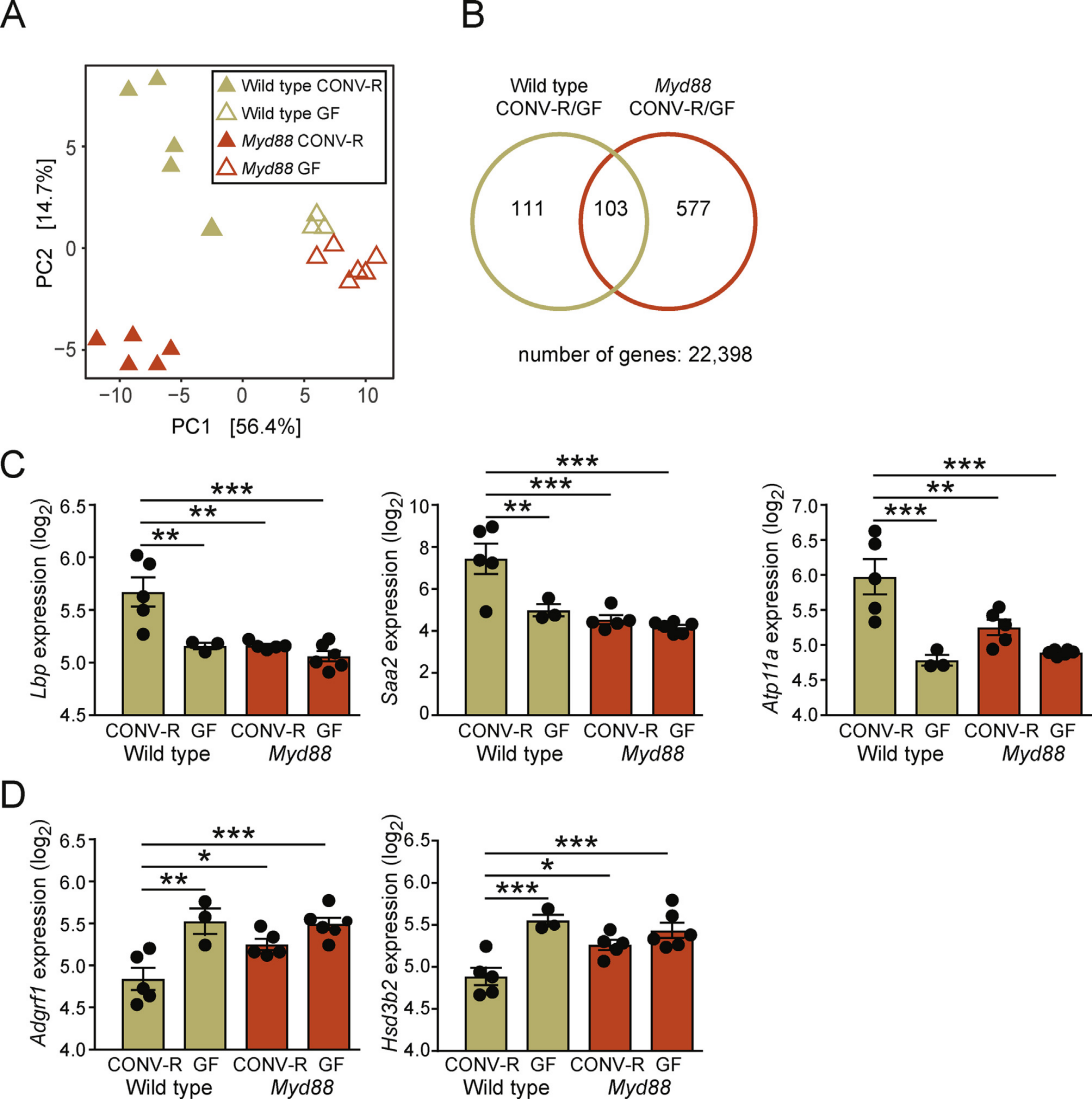
**Analysis of hepatic gene expression quantified by microarray in the CONV-R and GF wild-type and *Myd88* KO mice.** (A) Principle component analysis of transcriptional data. PC1, principal component 1; PC2, principal component 2. (B) Venn diagram showing the numbers of genes significantly regulated by the gut microbiota (at a 5% false discovery rate) in the wild-type mice (beige), *Myd*88 KO mice (red), and both strains (intersection between circles). Genes more than 2-fold upregulated (C) or downregulated (D) in the CONV-R wild-type mice compared to the CONVR *Myd*88 KO mice, GF wild-type mice, and GF *Myd*88 KO mice. Wild-type CONV-R, n = 5; wild-type GF, n = 3; *Myd88* CONV-R, n = 5; *Myd8*8 GF, n = 6. ∗P < 0.05, ∗∗P < 0.01, and ∗∗∗P < 0.001 determined by one-way analysis of variance and using Tukey's multiple comparisons test. Hierarchical multivariate statistical analysis of the genes presented in C-D resulted in the following P values: *Lbp*: P_micro_ = 0.00015, P_geno_ = 0.30, P_interaction_ = 0.0092; *Saa2*: P_micro_ = 0.00020, P_geno_ = 0.45, P_interaction_ = 0.0075; *Atp11a*: P_micro_ = 6.8e-06, P_geno_ = 0.044, P_interaction_ = 0.0045; *Adgrf1*: P_micro_ = 7.9e-05, P_geno_ = 0.047, P_interaction_ = 0.026; *Hsd3b2*: P_micro_ = 2.4e-06, P_geno_ = 0.10, P_interaction_ = 0.0063.

**Figure 2:**
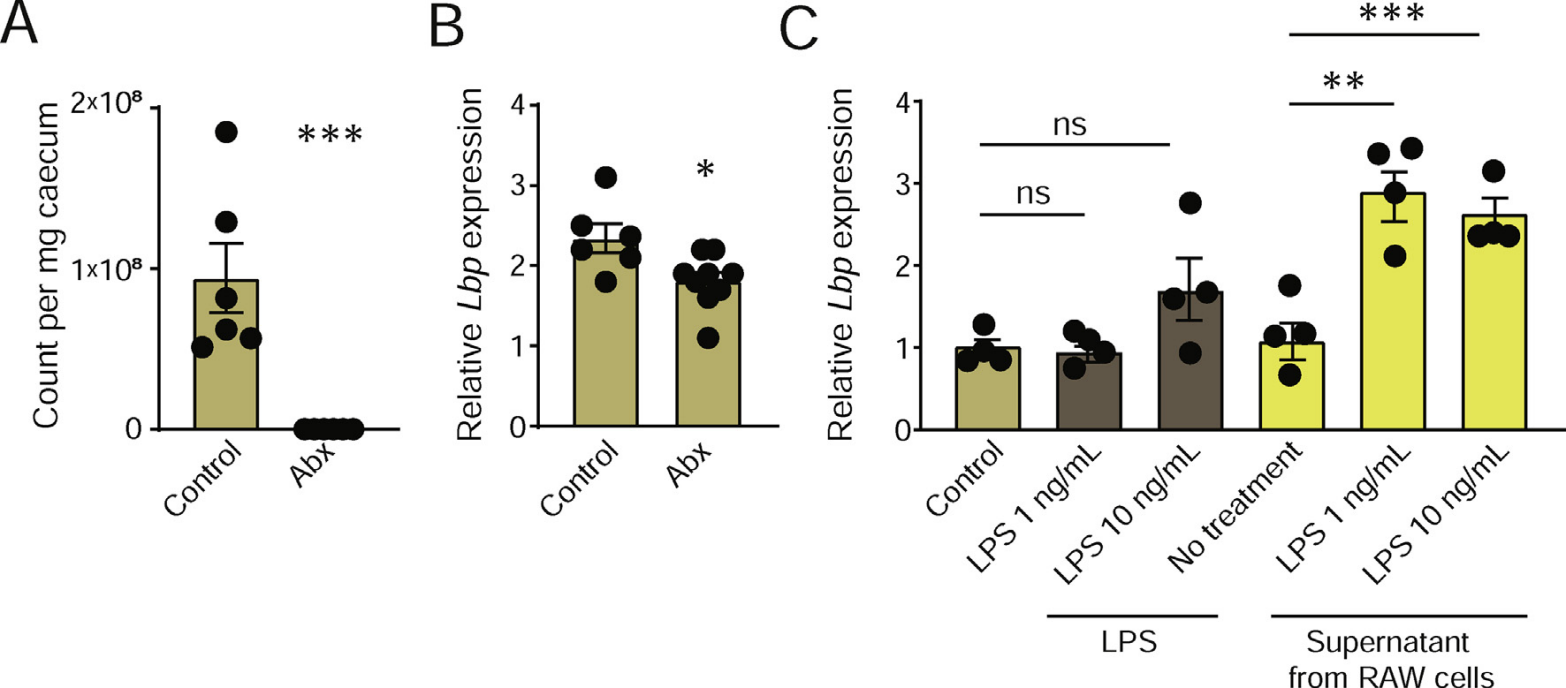
**Antibiotics treatment decreases hepatic *Lbp* expression and supernatant from RAW 264.7 macrophages pretreated with LPS induces *Lbp* expression in primary hepatocytes.** (A) Bacterial count in the cecum. (B) Hepatic *Lbp* expression. (C) Expression of *Lbp* in primary hepatocytes treated with LPS or supernatant from RAW 264.7 macrophages treated with LPS. n = 6–8 (A–B); n = 4 (C). ∗P < 0.05∗∗, P < 0.01, and ∗∗∗P < 0.001 determined by Student's t-test (A–B) or one-way analysis of variance and using Tukey's multiple comparisons test (C).

**Figure 3:**
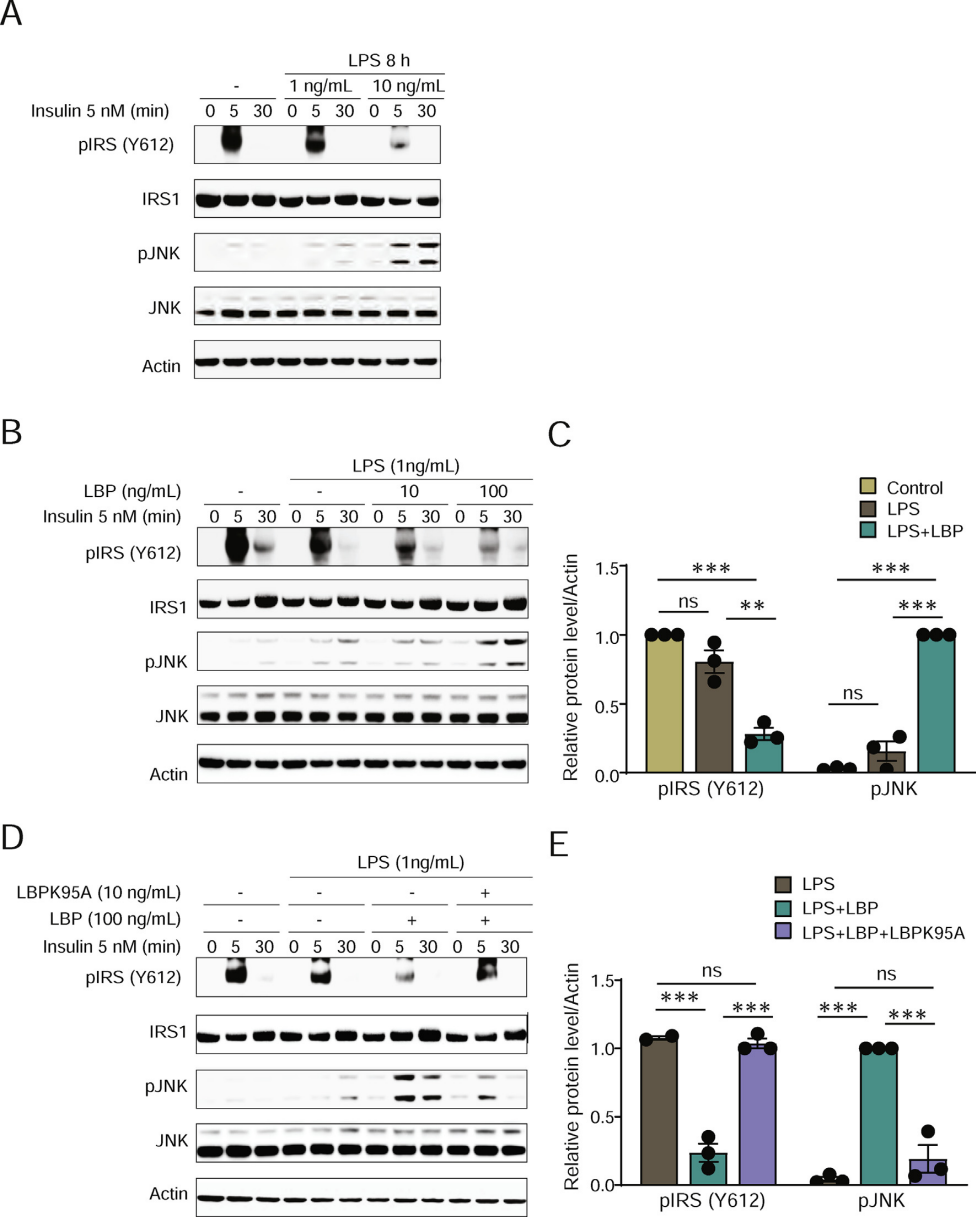
**Interaction between LPS and LBP increases JNK phosphorylation and decreases IRS1 phosphorylation in response to insulin in primary hepatocytes.** (A) Immunoblot showing the effect of LPS exposure on phosphorylation of IRS1 (Y612) and JNK in response to insulin. (B) Immunoblot showing the effect of LBP exposure on phosphorylation of IRS1 (Y612) and JNK in response to insulin in the presence of LPS. (C) Semi-quantitative analysis of the phosphorylation levels in panel B. (D) Immunoblot showing the effect of the LBP-blocking peptide LBPK95A on phosphorylation of IRS1 (Y612) and JNK in response to insulin in the presence of LPS and LBP. (E) Semi-quantitative analysis of the phosphorylation levels in panel D. n = 3. ∗∗P < 0.01 and ∗∗∗P < 0.001 determined by one-way analysis of variance and using Tukey's multiple comparisons test between all of the groups.

### 2.6 Western blotting

Snap-frozen tissues and harvested cells were lysed in buffer A containing 50 mM Tris–HCl (pH 7.4), 150 mM NaCl, 1 mM ethylenediaminetetraacetic acid, 1 mM Na3VO4, 20 mM NaF, 10 mM glycerophosphate, 1 mM phenylmethylsulfonyl fluoride (PMSF), 10% glycerol, 1% Triton X-100, and protease inhibitor cocktail. For Western blotting, the cell lysates were sonicated and centrifuged at 20,000 g for 15 min at 4 °C, and the supernatant was mixed with 5X Laemmli buffer, 0.156 M Trizma hydrochloride (pH 6.8), 25% glycerol, 12.5% β-mercaptoethanol, 12.5% sodium dodecyl sulfate (SDS), and 0.1% bromophenol blue followed by heating at 95 °C for 10 min. The samples (20–25 μg) treated with Laemmli buffer were separated on Bis-Tris gels, transferred to nitrocellulose membrane, and probed with the indicated antibodies. The blots were then reacted with horseradish peroxidase (HRP)-linked anti-rabbit IgG or anti-mouse IgG followed by enhanced chemiluminescence. Antibodies are reported in online Supplementary Table 1.

### 2.7 MRI, glucose tolerance, and insulin measurement

MRI, glucose tolerance tests, and measurements of insulin levels were conducted as previously described [4]. Briefly, glucose tolerance tests were performed by injecting glucose (2 g/kg body weight) intraperitoneally after a 5 h fast. Tail blood samples were collected at −30, 0, 15, 30, 60, 90, and 120 min and blood glucose levels were determined using a glucose meter (Accu Check Aviva, Roche). Insulin levels were measured with a Crystal Chem kit (Downers Grove, IL, USA) according to the manufacturers' protocols.

### 2.8 Immunohistochemistry of epididymal adipose tissue and liver

Paraffin-embedded epididymal adipose tissue and liver sections (7 μm) were processed as previously described [3]. Slides were stained with hematoxylin and quantitated by densitometric analysis using Biopix iQ software (version 2.1.3; Biopix, Sweden).

### 2.9 Statistical analysis

Statistical analyses were conducted using R and GraphPad Prism 7. Two-sided Student's t-tests were used to compare two groups. One-way ANOVA with Tukey's multiple comparison were used to compare three or more groups. For tests of four groups measuring the effect of two factors, 2-way ANOVA tests with Sidak's multiple comparisons were used. For tests between groups with repeated measurements, a 2-way ANOVA test for repeated measurement with Tukey's multiple comparison was used.

## 3 Results and discussion

### 3.1 Gut colonization status regulates the expression of hepatic genes related to LPS transport and cellular signaling through MYD88

The GF mice fed a chow diet had improved glucose tolerance [2,4] and reduced inflammation [3] compared to the CONVR mice. To investigate how colonization status affects the expression of genes associated with glucose metabolism through MYD88-dependent signaling, transcriptome analysis was conducted by microarray on liver samples from the GF and CONV-R wild-type and *Myd88* KO mice. To specifically assess the effect of bacterially derived inflammatory factors, we avoided the use of a high-fat diet since long-chain saturated fatty acids induce inflammation [26] and therefore may confound the signal.

Principle component analysis of gene expression showed that the mice separated on colonization status in the first dimension and genotype in the second dimension (Figure 1A). This was similar to what we previously observed for gene expression in the duodenum, ileum, and colon [16]. The GF mice of both genotypes clustered close together, suggesting that MYD88 is important for gut microbiota signaling in the liver. A biplot combining the PCA presented in Figure 1A with the underlying variables (genes) identified *Saa2* as the gene most strongly associated with CONV-R wild-type mice (Supplementary Figure 1). Among a total of 22,398 genes, 791 (3.5%) were regulated by colonization status (Figure 1B). To focus on microbially regulated functions dependent on MYD88, microbially regulated genes in the *Myd88* KO mice (*Myd88* KO and CONV-R/GF) were subtracted from microbially regulated genes in the wild-type mice (wild-type and CONV-R/GF), resulting in 111 genes (Figure 1B, beige circle, left section). These genes were enriched in the gene ontology (GO) categories LPS transport, sterol biosynthetic process, and regulation of ERK1 and ERK2 cascade (Supplementary Table 2). GO categories related to sterol metabolism were also regulated by colonization status in the *Myd88* KO mice (red circle, Figure 1B). Hence, only cellular processes related to LPS transport and cellular signaling were exclusively affected by colonization status in the wild-type mice. Next, to identify genes specifically and strongly regulated in the CONV-R wild-type mice, the dataset was filtered for genes that were more than 2-fold up- or downregulated in this group compared to all of the other groups. *Saa2*, *Lbp*, and *Atp11b* were found to be induced (Figure 1C) while *Adgrf1* and *Hsd3b2* were found to be suppressed (Figure 1D) in the CONV-R wild-type mice. Hierarchical multivariate analysis of the genes in Figure 1C–D (*Saa2*, *Lbp*, *Atp11b*, *Atp11a*, and *Adgrf1*) showed that colonization status had a significant (P < 0.05) impact on the expression of all genes while the genotype affected the expression of *Atp11a* and *Adgrf1.* The interaction between gut microbiota and genotype was significant for all of the genes.

Taken together, we showed that a group of hepatic genes mainly related to LPS transport and cellular signaling are regulated by gut colonization status through MYD88.

### 3.2 LBP is induced by the gut microbiota and inhibits insulin signaling in hepatocytes

Among genes identified as regulated by colonization status through *Myd88*, we chose to focus on *Lbp* since increased serum levels of LBP are strongly associated with impaired glucose homeostasis and type 2 diabetes in humans [[27], [28], [29], [30], [31], [32]]. LBP is an acute phase protein that facilitates immune signaling by presenting LPS to TLR4 [33], and the administration of LPS induces hyperglycemia, hyperinsulinemia, and insulin resistance in mice and humans [34,35].

To further investigate how the gut microbiota affects the expression of hepatic *Lbp*, we treated the mice with a broad-spectrum cocktail of antibiotics for 5 weeks. Antibiotics treatment depleted 99.9% of the bacteria in the cecum (Figure 2A). The expression of *Lbp* significantly decreased in the mice receiving antibiotics compared to the control mice (Figure 2B). This is in line with the observed decrease in *Lbp* expression in the GF mice (Figure 1C) and supports our hypothesis that *Lbp* may be regulated by the gut microbiota.

Next, to investigate putative mechanisms by which MAMPSs can induce *Lbp* expression, we treated primary hepatocytes with 1 ng/mL (physiological range level [4,34]) or 10 ng/mL LPS for 24 h. LPS treatment did not affect *Lbp* expression (Figure 2C). Acute phase proteins such as LBP produced by hepatocytes are known to be regulated by cytokines secreted from activated immune cells [36]. Hence, we treated the primary hepatocytes with supernatant from untreated RAW 264.7 macrophages or RAW 264.7 macrophages pretreated with 1 ng/mL or 10 ng/mL LPS. While supernatant from the untreated macrophages did not induce *Lbp* expression, supernatant from the macrophages pretreated with LPS significantly increased *Lbp* expression (Figure 2C). These results suggest that low levels of LPS can indirectly stimulate *Lbp* expression in hepatocytes through interactions with immune cells *in vitro.*

LPS exposure induces JNK phosphorylation [37] and insulin resistance [38] in hepatocytes. However, the LPS levels applied in *in vitro* and *in vivo* studies are usually higher than naturally occurring levels [15,35,38]. We thus investigated if LBP can potentiate the effect of LPS on insulin signaling in primary hepatocytes at low concentrations of LPS. First, we found that 10 ng/mL but not 1 ng/mL of LPS impaired insulin signaling by decreasing IRS-1 tyrosine phosphorylation (Y612) and increasing c-Jun N-terminal kinase (JNK) phosphorylation (Figure 3A). When cells were co-incubated with 1 ng/mL of LPS along with LBP, insulin-stimulated IRS1 phosphorylation decreased and JNK phosphorylation increased (Figure 3B,C). Next, to investigate if LBP potentiates the effect of LPS on insulin signaling via interaction between LPS and LBP, we co-incubated LPS and LBP with LBP-blocking peptide LBPK95A [17]. LBPK95A efficiently abolished the effect of LBP and restored insulin signaling (Figure 3D,E).

Taken together, these results show that LBP impairs insulin signaling in hepatocytes in the presence of low concentrations of LPS *in vitro*.

### 3.3 Systemic pharmacologic blocking or genetic downregulation of hepatic LBP improves glucose homeostasis

**Figure 4:**
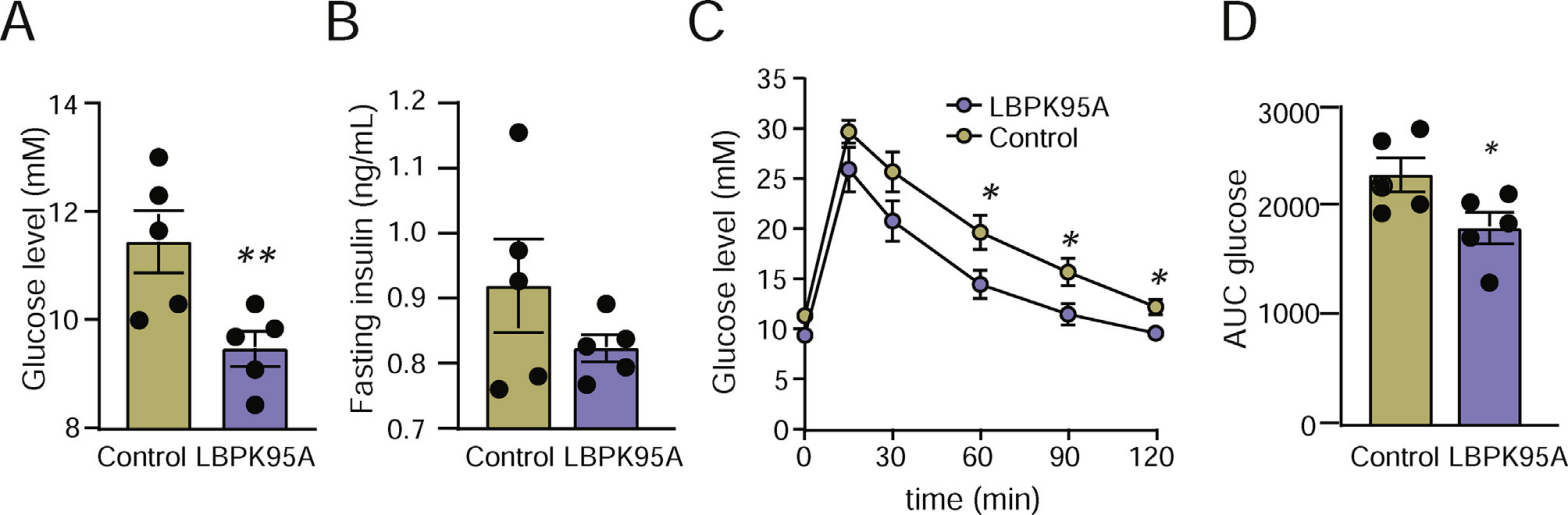
**Blocking of interaction between LPS and LBP with LBPK95A *in vivo* improves systemic glucose metabolism.** (A) Fasting glucose levels, (B) fasting insulin levels, and (C–D) glucose tolerance in the mice treated with LBPK95A or saline control. n = 5. ∗P < 0.05 and ∗∗P < 0.01 determined by Student's t-test.

To investigate how interaction between LPS and LBP affects glucose metabolism *in vivo*, we treated weight-matched CONV-R mice with LBPK95A or vehicle for 24 h. Fasting glucose levels decreased in the mice treated with LBPK95A compared to vehicle (Figure 4A), while the fasting insulin levels did not differ significantly between the groups (p = 0.2; Figure 4B). The mice treated with LBPK95A exhibited improved glucose tolerance compared to the mice receiving vehicle (Figure 4C–D). These data suggest that LBP can impair glucose homeostasis *in vivo*, possibly through interactions with LPS.

**Figure 5:**
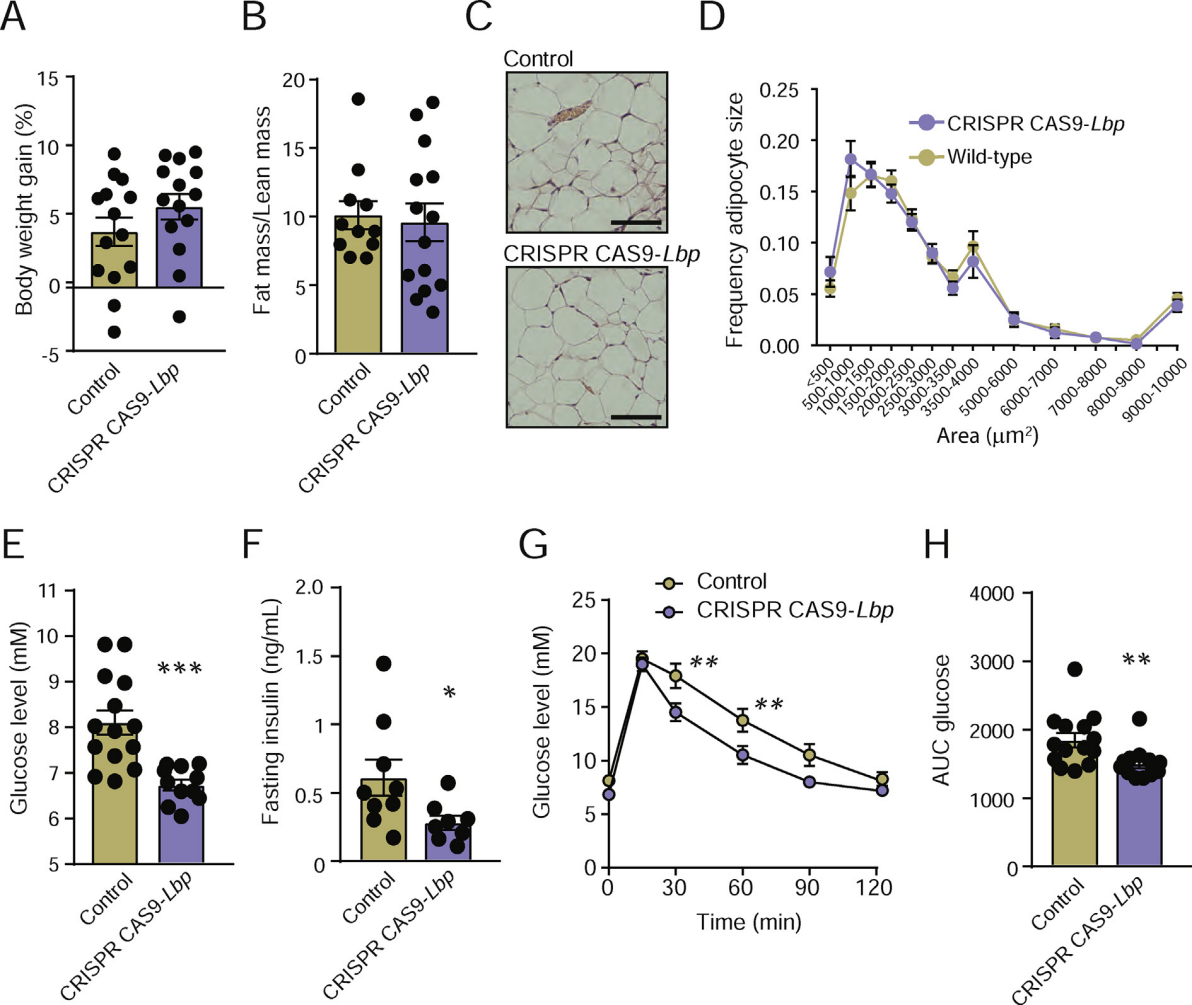
**CRISPR CAS9-mediated knockdown of *Lbp* improves systemic glucose metabolism.** (A) Body weight gain, (B) ratio between fat mass and lean mass, (C) representative hematoxylin staining of epididymal adipose tissue (scale bars = 100 μm), (D) frequency of adipocyte sizes, (E) fasting glucose levels, (F) fasting insulin levels, and (G–H) glucose tolerance in the mice treated with CRISPR CAS9-*Lbp* or adenovirus negative control. n = 14. ∗P < 0.05, ∗∗P < 0.01, and ∗∗∗P < 0.001 determined by Student's t-test.

*Lbp* is mainly expressed in the liver and adipose tissue [39]. While the present study demonstrated that the gut microbiota induces *Lbp* expression in the liver (Figure 1C), we previously showed that it does not affect expression in adipose tissue [24]. To investigate the link between microbial regulation of LBP and glucose homeostasis, we knocked down *Lbp* in the liver but not adipose tissue. The CONV-R mice were transduced with an adenovirus vector carrying a CRISPR/Cas9-construct targeting *Lbp* or a negative control vector. After 5 weeks, we conducted an intraperitoneal glucose tolerance test, and after 7 weeks, we sacrificed the mice. Consistent with previous reports showing that systemic delivery of recombinant adenovirus predominantly transduces genes into the liver [40] and not adipose tissue [41], Western blotting analysis revealed that the mice treated with CRISPR/Cas9-*Lbp* reduced hepatic LPB levels by approximately 50% compared to the mice receiving negative control vector, while the LBP levels in the epididymal adipose tissue were not affected by adenovirus treatment (Supplementary Figure 2). Body weight gain, fat mass/lean mass ratio, and adipocyte size did not differ between the treatment group and control group (Figure 5A–D). Neither did the levels of hepatic steatosis, inflammation, or fibrosis (Supplementary Figure 3). However, the mice treated with CRISPR/Cas9-*Lbp* had decreased fasting glucose and insulin levels (Figure 5E–F) and improved glucose tolerance compared to the control mice (Figure 5G–H).

Taken together, these results indicated that systemic blocking of LBP or decreased LBP levels in the liver improve glucose homeostasis, mainly by decreasing fasting glucose levels, but do not affect adiposity or steatosis.

Gavaldà-Navarro *et* *al.* reported that *Lbp* KO mice are leaner than wild-type mice but surprisingly also have impaired glucose metabolism [42]. The discrepancy to the *in vivo* models presented in our study may have several explanations. First, we used a blocking peptide to inhibit binding of LPS for 24 h. Features of the *Lbp* KO, such as increased adipose tissue browning [42], may take longer to develop. LBP may also have other functions apart from LPS binding that are not affected by the blocking peptide. For example, LBP has been shown to catalyze the exchange of phospholipids [43]. A direct link between interactions between LPS and LBP and glucose homeostasis was not previously demonstrated. Second, while Gavaldà-Navarro *et* *al.* studied the effect of whole-body knockout, our CRISPR-CAS9 model decreased LBP levels in the liver but not in adipose tissue. LBP has been shown to have a major impact on adipose tissue cell differentiation and energy balance [27,42,44]. Hence, the results from these models are not comparable. The metabolic features of an adipose tissue-specific *Lbp* KO remain to be investigated.

## 4 Conclusions

In summary, we showed that the gut microbiota increases the expression of *Lbp* in the liver through MYD88. We also demonstrated that the LBP-blocking peptide LBP95A improves insulin signaling in hepatocytes as well as systemic glucose metabolism. Furthermore, we showed that knockdown of LBP in the liver results in improved systemic glucose homeostasis. The present study demonstrated that inflammatory factors from the gut microbiota may affect glucose homeostasis independent of adiposity, and we suggest that LBP produced by hepatocytes may constitute a link between gut microbiota and glucose metabolism.

## Funding

This study was supported by several sources: 10.13039/501100001862Svenska Forskningsrådet` Formas (2017-01996_3 and 2017-02001), 10.13039/501100001674Fondation Leducq (17CVD01), the 10.13039/501100004359Swedish Research Council (Vetenskapsrådet), and grants from the Swedish state under an agreement between the Swedish government and the county councils, the ALF-agreement (ALFGBG- 718101). F.B. is a Torsten Söderberg Professor of Medicine and recipient of an ERC Consolidator Grant (10.13039/100010663European Research Council, Consolidator grant 615362-METABASE).
