# Supplementary material for: Hepatic expression of lipopolysaccharide-binding protein (Lbp) is induced by the gut microbiota through Myd88 and impairs glucose tolerance in mice independent of obesity
Source: Mol Metab. 2020 Apr 16;37:100997. doi: 10.1016/j.molmet.2020.100997 (PMC7229497; doi:10.1016/j.molmet.2020.100997)
Supplement: Multimedia component 1 [file mmc1.pdf]

SUPPLEMENTARY MATERIAL

1. Supplementary Figure 1
2. Supplementary Figure 2
3. Supplementary Figure 3
4. Supplementary Table 1
5. Supplementary Table 2

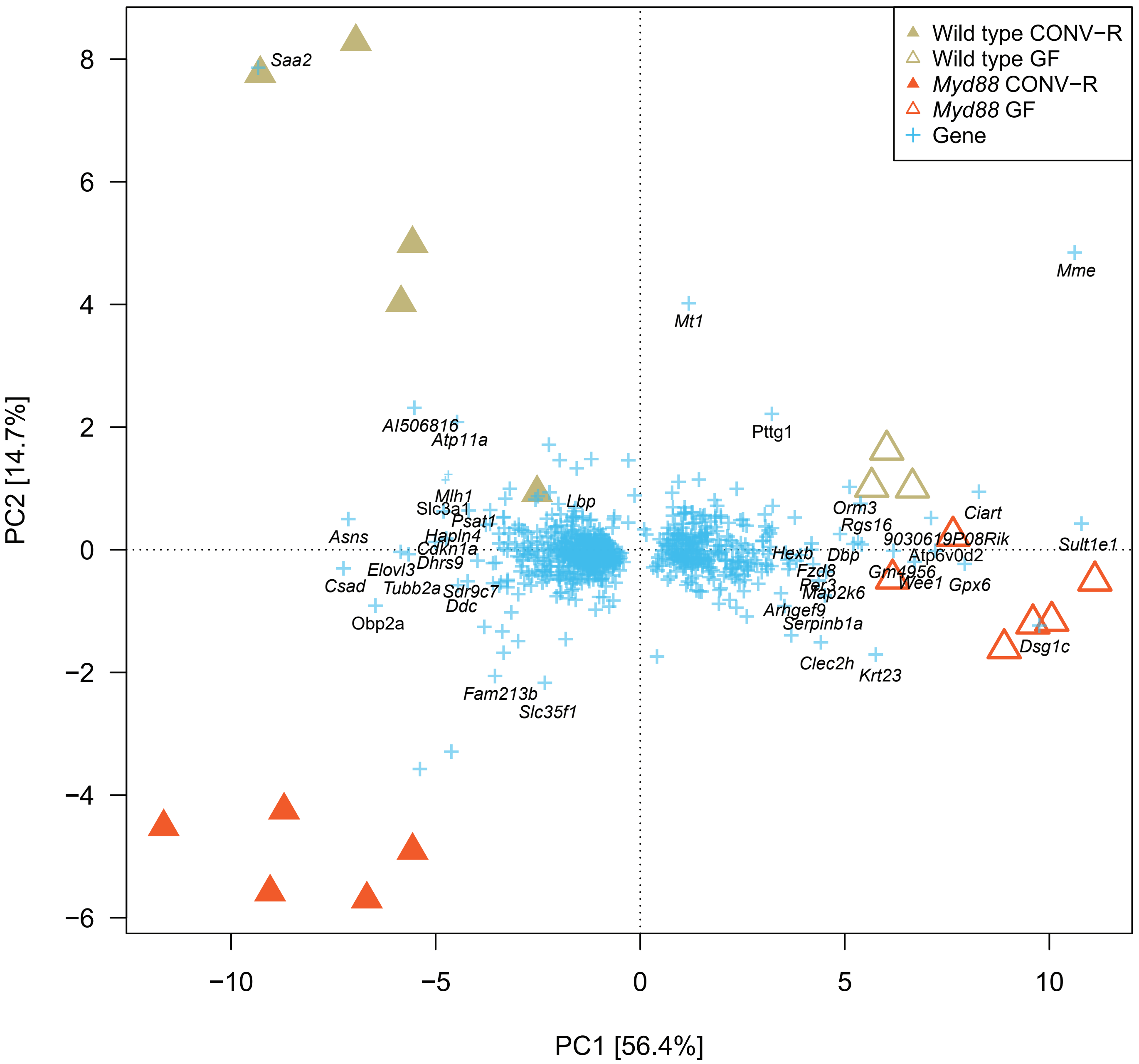

Supplementary Figure 1. Biplot of gene expression displaying PCA of samples and individual genes.

Supplementary Figure 1

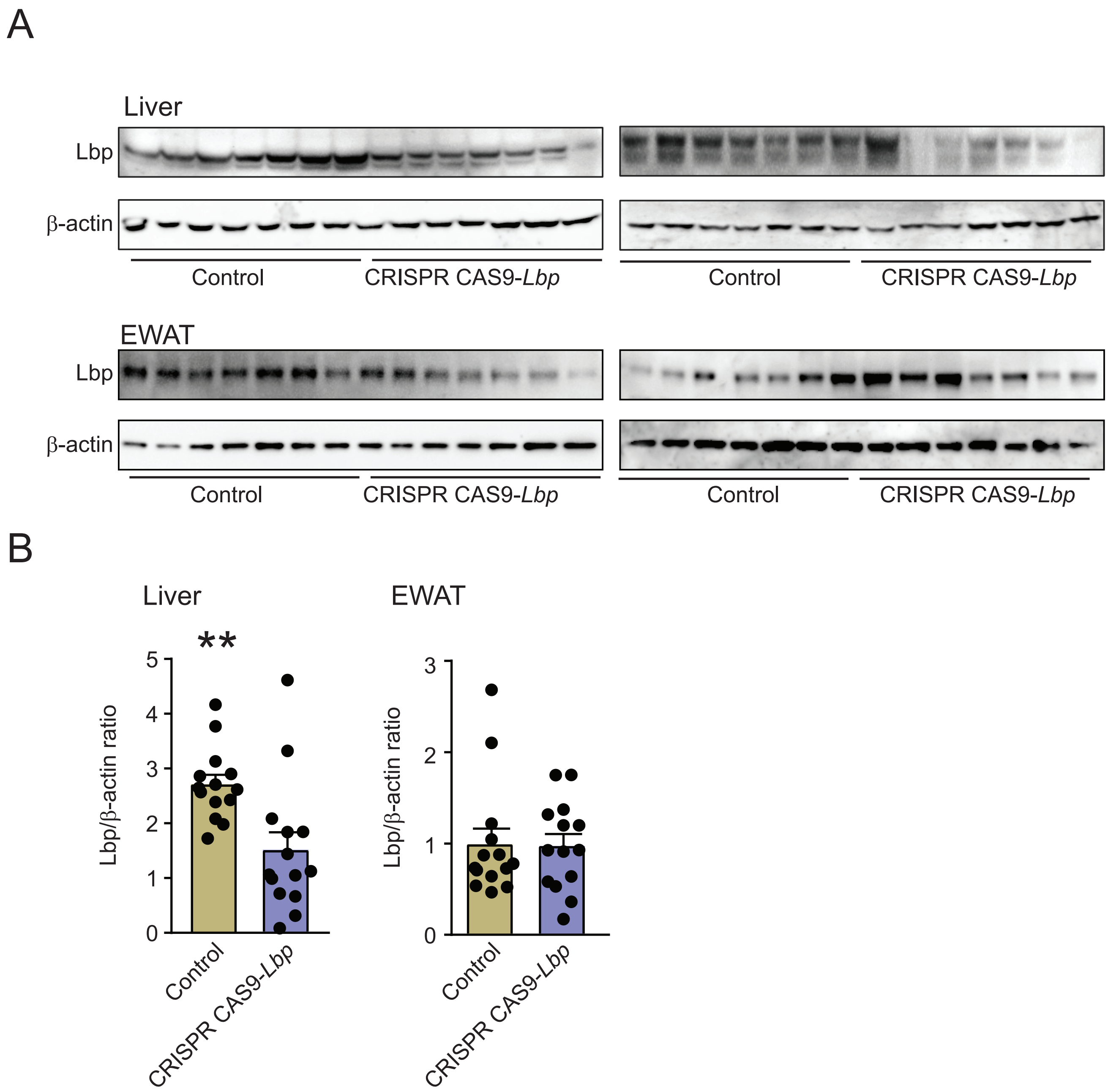

Supplementary Figure 2. Abundance of LBP in liver and adipose tissue in mice treated with CRISPR CAS9-*Lbp*. (A) Immunoblot showing LBP in liver and in adipose tissue from mice treated with adenovirus mediated CRISPR CAS9-*Lbp* or adenovirus negative control. (B) Semi-quantitative analysis of LBP abundance in liver and adipose tissue. n=14. **$p<0.01$ determined by Student's t-test.

Supplementary Figure 2

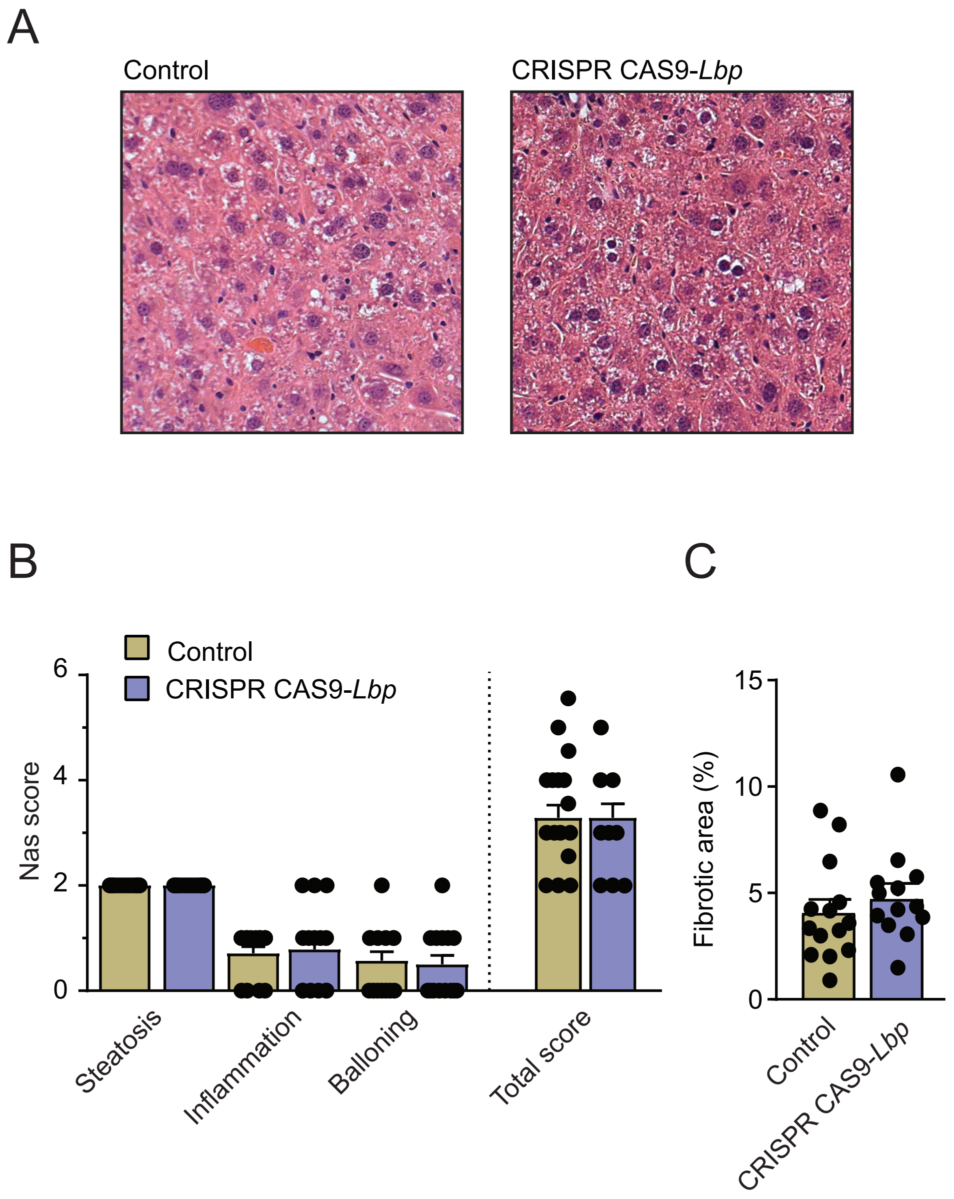

Supplementary Figure 3. Liver status in mice treated with CRISPR CAS9-*Lbp*. (A) Representative hematoxylin staining of liver tissue, (B) NAS score of liver tissue and (C) fibrotic area of liver tissue determined by Sirius Red staining in mice treated with adenovirus mediated CRISPR CAS9-*Lbp* or adenovirus negative control. n=14.

Supplementary Figure 3

Supplementary Table 1. Antibodies used in the study.

Antibody	Manufacturer	Product number
Rabbit anti-β-Actin (13E5) (1:500)	Cell Signaling	4970
Rabbit anti-IRS1 (1:500)	EMD Millipore	06-248
Rabbitanti-phospho-IRS1 (Tyr612) (1:500)	EMD Millipore	09-732
Rabbit anti-phospho-JNK (Thr183/Tyr185) (1:500)	Cell Signaling	4668
Mouse anti-JNK1 (1:500)	Cell Signaling	3708
Sheep anti-LBP (1:500)	R&D Systems	AF6635

Supplementary Table 2. Biological processes in the liver regulated by the gut microbiota in wild type and *MyD88* KO mice.

**Category**	**Biological process**	**p-value**	
		**Wild type**	**MyD88**
**Regulated wild type**			
GO:0015920	lipopolysaccharide transport	3.27E-02	
GO:0016126	sterol biosynthetic process	3.88E-05	
GO:0070372	regulation of ERK1 and ERK2 cascade	8.89E-03	
**Regulated wild type and MyD88**			
GO:0006629	lipid metabolic process	9.06E-07	8.65E-13
GO:0006730	one-carbon metabolic process	9.63E-04	2.24E-04
GO:0006732	coenzyme metabolic process	3.27E-02	4.42E-08
GO:0006790	sulfur compound metabolic process	9.61E-03	6.24E-06
GO:0008152	metabolic process	3.00E-05	5.74E-13
GO:0008202	steroid metabolic process	9.06E-07	4.60E-05
GO:0016125	sterol metabolic process	1.64E-06	6.82E-03
GO:0042445	hormone metabolic process	1.75E-02	2.19E-02
GO:0044237	cellular metabolic process	3.67E-03	1.44E-13
GO:0044238	primary metabolic process	4.39E-02	9.55E-09
GO:0044281	small molecule metabolic process	1.06E-07	7.42E-26
GO:0044283	small molecule biosynthetic process	7.20E-05	5.09E-09
GO:0044699	single-organism process	5.51E-03	1.47E-03
GO:0044710	single-organism metabolic process	2.76E-10	1.03E-24
GO:0044711	single-organism biosynthetic process	9.96E-05	4.73E-09
GO:0051186	cofactor metabolic process	1.46E-02	1.27E-06
GO:0051923	sulfation	8.89E-03	1.25E-02
GO:0055114	oxidation-reduction process	9.96E-05	6.72E-15
GO:0071704	organic substance metabolic process	1.36E-02	2.06E-10
GO:1901564	organonitrogen compound metabolic process	7.21E-03	8.63E-12
GO:1901615	organic hydroxy compound metabolic process	6.02E-06	6.14E-04
**Regulated MyD88**			
GO:0005975	carbohydrate metabolic process		6.77E-03
GO:0006575	cellular modified amino acid metabolic process		2.18E-06
GO:0006706	steroid catabolic process		1.25E-03
GO:0006760	folic acid-containing compound metabolic process		1.35E-02
GO:0006766	vitamin metabolic process		2.40E-02
GO:0006793	phosphorus metabolic process		2.33E-03
GO:0006807	nitrogen compound metabolic process		1.39E-04
GO:0007586	digestion		2.12E-02
GO:0008209	androgen metabolic process		1.93E-02
GO:0009056	catabolic process		4.30E-09
GO:0009058	biosynthetic process		1.31E-03
GO:0009069	serine family amino acid metabolic process		1.42E-02
GO:0009437	carnitine metabolic process		4.36E-02
GO:0009991	response to extracellular stimulus		6.21E-03
GO:0010033	response to organic substance		3.28E-05
GO:0010817	regulation of hormone levels		4.15E-03
GO:0010876	lipid localization		1.09E-02
GO:0019373	epoxygenase P450 pathway		7.48E-05
GO:0019915	lipid storage		6.34E-05
GO:0032787	monocarboxylic acid metabolic process		7.16E-14
GO:0032922	circadian regulation of gene expression		3.56E-03
GO:0034330	cell junction organization		1.12E-02
GO:0034641	cellular nitrogen compound metabolic process		3.17E-02
GO:0042180	cellular ketone metabolic process		2.54E-05
GO:0042221	response to chemical		1.68E-04
GO:0042398	cellular modified amino acid biosynthetic process		4.83E-02
GO:0042558	pteridine-containing compound metabolic process		6.01E-03
GO:0044712	single-organism catabolic process		1.02E-11
GO:0044723	single-organism carbohydrate metabolic process		1.79E-02
GO:0045216	cell-cell junction organization		1.20E-02
GO:0046320	regulation of fatty acid oxidation		1.27E-06
GO:0046942	carboxylic acid transport		1.55E-05
GO:0050892	intestinal absorption		2.53E-04
GO:0051179	localization		1.20E-02
GO:0051341	regulation of oxidoreductase activity		4.06E-02
GO:0051353	positive regulation of oxidoreductase activity		8.24E-03
GO:0055086	nucleobase-containing small molecule metabolic process		5.62E-04
GO:0065008	regulation of biological quality		1.62E-04
GO:0071702	organic substance transport		2.78E-02
GO:0072524	pyridine-containing compound metabolic process		3.06E-02
GO:0072593	reactive oxygen species metabolic process		6.77E-03
GO:0090322	regulation of superoxide metabolic process		1.70E-02
GO:1901135	carbohydrate derivative metabolic process		4.86E-04
GO:1901360	organic cyclic compound metabolic process		9.29E-03
GO:1901566	organonitrogen compound biosynthetic process		5.28E-03
GO:1901576	organic substance biosynthetic process		2.14E-03
GO:1901657	glycosyl compound metabolic process		4.69E-02
GO:2000323	negative rgulation of glucocorticoid receptor signaling pathway		4.50E-02
